# Connecticut Medical Society

**Published:** 1846-08

**Authors:** 


### CONNECTICUT MEDICAL SOCIETY.

We have received a neat, well printed pamphlet of 36 pages entitled "Proceedings at the Annual Convention of the Connecticut Medical Society, May, 1846, together with a list of Members and the Annual Address." The officers of this Society are, Archibald Welch, M.D., Presdent; Dyer T. Brainard, M.D., Vice President; V. M. Dow, M.D., Treasurer; Gurdon W. Russel, M.D., Secretary. The Society has been in existence half a century, and we learn that as long ago as 1794 a paper was read by Dr. S. H. P. Lee, at one of its meetings, on *Autumnal Bilious Fever.* From 1804 to 1817 there seems to have been an intermission in its labors, since no dissertation is reported as having been read during that time. The Society at its late meeting passed resolutions highly complimentary to the Medical Institution of Yale College. The annual address was delivered to the Society by Theodore Sill, M. D., the subject being typhoid fever. Every one who feels an interest in the progress of the medical profession must be gratified at the spirit exhibited by the Societies which now annually hold their meetings in so many States in the Union. Where there is so much united, well directed effort, there will be results. A more liberal, fraternal feeling is awakened among the members, and the impulses of a generous ambition are quickened in all the ranks of the profession.

